# Tropical land-use change alters trait-based community assembly rules for dung beetles and birds

**DOI:** 10.1007/s00442-020-04829-z

**Published:** 2021-02-08

**Authors:** Felicity A. Edwards, David P. Edwards, Keith C. Hamer, Tom M. Fayle

**Affiliations:** 1 School of Biology, University of Leeds, Leeds, LS2 9JT UK; 2 Department of Animal and Plant Sciences, University of Sheffield, Sheffield, S10 2TN UK; 3 Biology Centre of the Czech Academy of Sciences, Institute of Entomology, Ceske Budejovice, 370 05 Czech Republic; 4 Institute of Tropical Biology and Conservation, Universiti Malaysia Sabah, Kota Kinabalu, Sabah Malaysia

**Keywords:** Avian, Borneo, Functional traits, Scarabaeidae, Species co-occurrence

## Abstract

Tropical rainforest disturbance and conversion are critical drivers of biodiversity loss. A key knowledge gap is understanding the impacts of habitat modification on mechanisms of community assembly, which are predicted to respond differently between taxa and across spatial scales. We use a null model approach to detect trait assembly of species at local- and landscape-scales, and then subdivide communities with different habitat associations and foraging guilds to investigate whether the detection of assembly mechanisms varies between groups. We focus on two indicator taxa, dung beetles and birds, across a disturbance gradient of primary rainforest, selectively logged rainforest, and oil palm plantations in Borneo, Southeast Asia. Random community assembly was predominant for dung beetles across habitats, whereas trait convergence, indicative of environmental filtering, occurred across the disturbance gradient for birds. Assembly patterns at the two spatial scales were similar. Subdividing for habitat association and foraging guild revealed patterns hidden when focusing on the overall community. Dung beetle forest specialists and habitat generalists showed opposing assembly mechanisms in primary forest, community assembly of habitat generalists for both taxa differed with disturbance intensity, and insectivorous birds strongly influenced overall community assembly relative to other guilds. Our study reveals the sensitivity of community assembly mechanisms to anthropogenic disturbance via a shift in the relative contribution of stochastic and deterministic processes. This highlights the need for greater understanding of how habitat modification alters species interactions and the importance of incorporating species’ traits within assessments.

**Supplementary Information:**

The online version contains supplementary material available at 10.1007/s00442-020-04829-z.

## Introduction

Habitat modification via selective logging and forest conversion to agriculture is widespread across the tropics (Asner et al. 2009; Gibbs et al. 2010), with agricultural expansion being the leading cause of the on-going global extinction crisis (Maxwell et al. 2016). Research on the impacts of tropical land-use change on biodiversity has focused extensively on how communities are affected in terms of species richness, composition, and functioning (e.g., Barlow et al. 2007; Gibson et al. 2011). These studies have revealed that selectively logged forests often have high conservation value and that conversion of forest to agriculture greatly reduces that value (Slade et al. 2011; Putz et al. 2012; Edwards et al. 2014a). However, much less attention has been given to understanding how tropical land-use change impacts patterns of community assembly.

The complexity of lowland tropical ecosystems, especially in the face of anthropogenic disturbance, represents a significant challenge to unravelling the contribution of assembly mechanisms—environmental filtering, limiting similarity, or stochasticity—in maintaining hyper-biodiversity. The interplay between species colonisation, local extinction, and shifts in dominance alter in response to changing biotic resources, microclimates (or other abiotic conditions), and interactions with other species that are characteristic of anthropogenic disturbance (Tylianakis et al. 2008; Sanders et al. 2003; Mori et al. 2018). Consequently, such disturbances could alter or remove ecosystem processes and functions (Bregman et al. 2015).

Previous work has largely inferred species assembly mechanisms from patterns of species co-occurrence (Gotelli and McCabe 2002). However, it is often unclear whether segregation is due to direct competition between ecologically similar species (‘niche-based assembly’) or environmental filtering of ecologically different species. Incorporating functional traits (e.g. bill dimensions) can detect whether species sharing traits co-occur infrequently, suggesting resource competition, or co-occur more commonly, inferring environmental filtering (Dayan and Simberloff 2005). Investigating community-assembly patterns within a functionally or environmentally determined subset of a community can subvert this issue by distinguishing if the variation between subsets is masked by overall assembly patterns (i.e. across all functions and environments; Bergman et al. 2015). A key question, therefore, is how land-use change influences co-occurrence when applying these approaches.

Interpreting the strength of different assembly mechanisms in driving co-occurrence patterns is inherently scale dependent (Swenson et al. 2007; Perronne et al. 2017). For example, niche differentiation occurs at fine spatial scale via limiting similarity and competitive dominance (e.g. 1 cm to ~ 10 m for plants; Götzenburger et al. 2012), whereas environmental filtering frequently occurs at medium- to landscape-scales encompassing a broader range of abiotic processes, such as soil type or climatic variables (de Bello et al. 2013). Environmental filtering can also occur at finer scales in highly heterogeneous environments (e.g. forest vs adjacent road; Adler et al. 2013; Bergholz et al. 2017).

Understanding the dynamics of community assembly is critical for conservation strategies aiming to protect and restore tropical forests (Mayfield and Levine 2010; de Bello et al. 2012; Fitzgerald et al. 2017; Hung et al. 2019). For example, communities driven by environmental filtering and competition (driven by limited resources) will potentially benefit more from conservation management focused on improving habitat quality and structure (Wearn et al 2018). Previous research has revealed significant impacts of tropical habitat modifications on community assembly of ants, understorey plants and small mammals in Malaysia (Fayle et al. 2013; Döbert et al. 2017; Wearn et al. 2018), dung beetles in Brazil (Audino et al. 2017), and trees in China (Ding et al. 2012). Linking these species’ functional traits to co-occurrence has revealed trait clustering of understory plants in selectively logged (and intact) tropical forest indicating potential environmental filtering, while salvage logging resulted in random trait assembly (Döbert et al. 2017). Tree communities (saplings, treelets and adult trees) show opposing patterns across development stages and scales: random trait assembly and trait divergence were predominant in old-growth forests, logged forest communities showed frequent trait divergence, while following shifting agriculture trait convergence was common (Ding et al. 2012). Thus, how assembly mechanisms of invertebrates and birds, as key components of tropical fauna, are impacted by logging and deforestation, and how such patterns are impacted by spatial scale are key remaining knowledge gaps.

We focus on dung beetles and birds to compare community assembly patterns across an anthropogenic disturbance gradient of primary forest, selectively logged forest and oil palm plantation in Sabah, Malaysian Borneo. Both taxa are reliable indicators of the wider ecosystem condition and function, and have well-documented trait information that relates directly to the key ecosystem functions they provide (Gardner et al. 2008; Flynn et al. 2009; Nichols et al. 2008; Slade et al. 2007). Dung beetles are notable ecosystem engineers, vital in nutrient recycling, secondary seed dispersal and soil structure, while birds are critical to pollination and seed dispersal networks. Both taxa retain community-level functional diversity after logging and suffer major reductions after conversion to oil palm (Edwards et al. 2013, 2014b), but differences in the assembly mechanisms operating between habitats remain unexplored. Here, we use a trait-based null modelling approach to explore how species co-occur within habitats and across a disturbance gradient in relation to functional trait similarity. We address three key questions: (1) How do non-random assembly mechanisms vary with habitat disturbance and spatial scale (local and landscape); (2) Do assembly mechanisms vary with the degree of habitat specialisation; and (3) Do assembly mechanisms vary between dung beetle nesting and avian feeding guilds?

## Materials and methods

### Study location

This study was conducted within the one million ha Yayasan Sabah (YS) logging concession in eastern Sabah, Malaysian Borneo (4° 58ʹ N, 117^o^ 48ʹ E; Online Resource 1). The majority of the concession (~ 90%) has been selectively logged, primarily between the 1970s and 2008 across two rotations of logging (for further details see Reynolds et al. 2011). Within the YS concession are ~ 130,000 ha of primary forest (Danum Valley Conservation Area, Palum Tambun Watershed Reserve, Maliau Basin, Imbak canyon and adjacent Virgin Jungle Reserves), and adjacent are extensive oil palm plantations (over 1,500,000 ha land coverage in Sabah, Reynolds et al. 2011).

### Sampling

Fieldwork took place from May to August 2008, May to October 2009 and February to September 2011, corresponding with the drier season each year. Across the study area, we sampled primary forest, once-logged forest, twice-logged forest and oil palm plantations (Online Resource 1b). Sampling effort was equalised across habitat types for dung beetles and birds (data from Edwards et al. 2011, 2014a) [*n*_Sites_ = 4 (landscape scale) across all four habitats]. Each site consisted of one line transect for bird sampling and two-line transects (a minimum of 500 m apart) for dung beetle trapping (see below for further methods). Sampling for birds and dung beetles occurred at the same sites across the forested habitats and at three of the oil palm sites, however the fourth oil palm site, for each taxa, was sampled in different locations due to logistical reasons (thus overall 17 sites were sampled but only 16 sites per taxa) (Online resource 1a). Sampling within oil palm was restricted to mature plantations (10–15 years old). The environmental conditions across sampling years remained similar (i.e. no mast-fruiting, droughts or floods). Primary forests are heterogeneous in structure with a dense canopy, extended vertical strata, and an open understorey with low densities of lianas when compared to logged forests (Magrach et al. 2016). Logged forests often have a significantly lowered canopy, higher densities of lianas, more tree fall gaps, and have numerous *‘new’* microhabitats formed from logging activities such as water pools, skid lines/small logging roads, and bare ground. Oil palm plantations are more uniform, have minimal understorey, with compacted soil and higher temperatures (Luke et al. 2019).

Dung beetles (Coleoptera: Scarabaeidae: Scarabaeinae) were sampled using standardised baited pitfall traps across all habitats [*n*_Microsites_ = 160, one pitfall trap per microsite (local scale); Online Resource 1c; following Edwards et al. 2011]. Pitfall traps baited with human dung were set at 100 m intervals to ensure independence (Larsen and Forsyth 2005). A single pitfall trap represents an individual microsite, and a site consisted of 10 pitfall traps. Traps were set for four days and re-baited after 48 h, with dung beetles collected every 24 h, and stored in ethanol. Individuals were identified to species level using reference collections (T. Larsen), which are housed at the Forest Research Centre, Sandakan, Malaysia and Smithsonian Museum, Washington DC, USA.

Unlimited-radius point counts were used to sample birds across all habitats [*n*_Microsites_ = 192 (local scale); Online Resource 1c; following Edwards et al. 2011]. Point count stations were set at 250 m intervals to ensure independence (Lees and Peres 2006). All birds that were heard or seen were recorded, and each station was visited for 15 min on each of three consecutive days. Point counts were run between 06:00 and 09:30 across each day, and where possible the ordering of the points was mixed up. The highest count for each species across the three days was classified as the final abundance for a given species, due to the high site fidelity of many tropical birds (Edwards et al. 2011). A single point count represents an individual microsite, and a site consisted of 12 point count stations (3 km transect length per site).

### Data analysis

#### Functional traits

Functional traits that reflect the key functional roles of dung beetles and birds were assessed for use with trait assembly null models described below. We combined both behavioural (nesting guild, diet range, diel activity, foraging strategy, mode, and substrate) and morphological (body size and bill structure) traits to capture a greater proportion of the variation across species as per Edwards et al. (2013, 2014b, respectively) (see Online Resource 2 for further details). We used the ‘dist.ktab’ function within the ‘ade4’ package in R (Dray and Dufour 2007) to create a dissimilarity matrix using Gower’s coefficient of distance where all traits were equally weighted. This function also allows for varying trait types, including multi-choice binary traits where a species can belong to more than one sub-group (e.g. a bird can be an insectivore, frugivore and nectivore). Functional analyses were performed using the ‘dbFD’ function of the ‘FD’ package in R (Laliberté et al. 2014).

#### Null models to test for overall community assembly mechanisms

To assess how functional traits and species abundances influence community assembly, we used a null model approach to test for trait divergence or convergence across our disturbance gradient (Bergholz et al. 2017; Fitzgerald et al. 2017; Dobert et al. 2017). Using the dissimilarity matrix calculated above and species abundances, we calculated the observed value of Rao’s quadratic entropy (RaoQ) (Online resources 3–10), a metric that describes the functional divergence and functional richness of a community (Botta-Dukát 2005; Mouchet et al. 2010). Observed RaoQ specifically measures the average difference across all measured traits for two randomly selected individuals from the community (Botta-Dukát 2005). These observed RaoQ results support previous work (Edwards et al. 2013, 2014b), showing that functional diversity alters across this disturbance gradient, with greater shifts after conversion to oil palm compared to disturbance from selective logging (Online resource 3–6).

To then be able to assess community assembly patterns across our disturbance gradient we compared the deviation of observed RaoQ from the expected null distribution, using the standardised effect size (SES) (SES_RaoQ), taking the approach of Gottelli and McGabe (2002). SES_RaoQ_ is defined as: $\left(\frac{\mathrm{Observed RaoQ}-\mathrm{mean null RaoQ}}{\mathrm{SD null RaoQ}}\right)$. Observed RaoQ is calculated using raw species data, while the mean and standard deviation (SD) of null RaoQ are generated from simulated null models (described below). This technique evaluates whether observed RaoQ differs significantly from what would be expected if species co-occurred at random, i.e. whether species were more or less functionally similar than expected by chance (Plass-Johnson et al. 2016), and is frequently used in assessing community assembly mechanisms (Perronne et al. 2017). This null modelling approach accounts for changes between habitats in species richness, species abundance and trait distributions (all of which can affect observed community functional diversity) allowing an assessment of trait-based species co-occurrence. SES_RaoQ also specifically has greater power in detecting community assembly mechanisms compared to other indices (Mason et al. 2013). We evaluated SES_RaoQ_ for each habitat separately at the local (*n*_dung beetles_ = 40 and *n*_birds_ = 48) and landscape (*n* = 4) scale (Online resource 1), and tested whether SES_RaoQ_ values were significantly different from zero (indicating a random trait distribution) using a student’s *t*-test.

The detectability of assembly mechanisms can be highly variable depending on the chosen randomisation algorithm (Götzenburger et al. 2016; Bernard-Verdier et al. 2012; Perronne et al. 2017) and is known to be scale dependent (Swenson et al. 2007; Perronne et al. 2017). To account for these two issues, we ran two complementary null models with different randomisation algorithms (described below), applying each null model at both local (dung beetles: *n*_Microsites_ = 40 per habitat; birds: *n*_Microsites_ = 48 per habitat) and landscape (*n*_Sites_ = 4 per habitat) scales. Therefore, for any given subset of the community (see variations of community subsets below), four models were run for each taxa. All null model randomisations were run with 10,000 permutations and with abundance data, which has been shown to maximise detection power (Götzenburger et al. 2016).

First, we used a ‘richness’ randomisation algorithm, which randomises the abundances of species between all co-occurring species in a sample (*model 1*). This model has the effect of removing any relationship between the traits and abundances of species co-occurring at a sampling site while maintaining the sample species richness and the total sample abundance (Kembel et al. 2010). Removing the link between trait values and abundances gives *model 1* particularly good detection of limiting similarity while fixing total sample abundance is critical as competition is inherently linked to individual abundances, i.e., a competitively strong species will increase in abundance and vice versa (Götzenburger et al. 2016). Second, we considered a ‘frequency’ randomisation algorithm, which randomised abundances within each species across all sites sampled (even those at which a species was absent in the observed dataset) (*model 2*). Importantly, this maintains the link between species abundances and traits (thus differing from *model 1*), while also maintaining species frequencies and total abundances, but allowing sample abundance and richness to vary (Kembel et al. 2010). A frequency model has stronger power to detect environmental filtering than models that fix the position of species in a matrix (i.e. models that only vary the abundances of species) (Götzenburger et al. 2016). Both these randomisation algorithms minimise type 1 errors (especially using abundance data) and show strong power and detectability for assembly mechanisms (Götzenburger et al. 2016).

Analyses were performed using the randomize Matrix function in the ‘picante’ package in R where these null models are standard options, specifically the *null.model* argument in the function is specified as ‘richness’ for our model 1 and ‘frequency’ for our model 2 (Kembel et al. 2010), in R v.3.6.1 (R Core Team 2019). We interpreted the model outputs as follows. Trait convergence is inferred when species exhibit similar traits (RaoQ observed < RaoQ expected; negative SESRaoQ). Trait divergence is inferred when species have more distinct traits (RaoQ observed > RaoQ expected; positive SESRaoQ). A random co-occurrence is inferred when observed values are close to expected values (SESRaoQ is around zero).

#### Co-occurrence in relation to habitat association

To explore whether any changes in co-occurrence patterns related to changes in interactions determined by species’ habitat associations, we tested whether assembly mechanisms differed between those species shared across all habitats (habitat generalists) and those that were not (non-shared). Habitat generalists were defined as those species found in all four habitats (*n* = 15 dung beetles, *n* = 21 birds). Habitat specialists were defined as those species that were unique to either oil palm (*n*_dung beetles_ = 25, *n*_birds_ = 20) or forested habitats (*n*_dung beetles_ = 40, *n*_birds_ = 159) (i.e. species found in at least one forest type but not oil palm). We analysed both subsets of species using the same null models at both the local and landscape scales. To ensure our definition of habitat association was not influenced by rare species (i.e. rare species could be rare across additional habitats where we did not record them thus giving a false value of habitat specialisation), we re-analysed these models with singletons removed. The results mirrored those from the full community; we, therefore, present only the full community results in the main text and provide both model outputs in the supplementary material (Online resource 5).

#### Co-occurrence in relation to nesting and foraging guilds

To test whether any changes in co-occurrence patterns were related to changes in guild representation across habitats, we analysed species by dominant guilds for both dung beetles and birds. Dung beetles were separated into distinct nesting/foraging strategies, while birds were separated into distinct foraging guilds. These guilds were chosen due to the functional relevance and the kinds of data available. We focused on dung beetle species that either tunnel or roll dung away from a resource. The single dung dweller species represented in our dataset were removed from these analyses. Rollers are absent from oil palm and are therefore only analysed across forest habitats. For birds, we used the dominant foraging guilds (Diet-5Cat) described in the Elton Trait database, which are based on the summed proportion of five individual diet components for each species (Wilman et al. 2014). In our dataset Plant/Seed and Vertebrate/Fish/Scavenger species were not represented by sufficient numbers of species (number of species must be greater than the number of traits to calculate RaoQ) in a given habitat to allow analyses to be run and thus were removed. Our analyses, therefore, focused on three groups: frugivores/nectivores, insectivores, and omnivores. Specifically, we tested whether those species from different guilds differed in their assembly mechanisms across the habitats. To do this, we analysed these subsets of species using the same null models described above at both the local and landscape scales. The specific trait being analysed was excluded from the functional analyses since this took the same value for all species in each of these analyses.

## Results

### Overall community assembly mechanisms

**Fig. 1:**
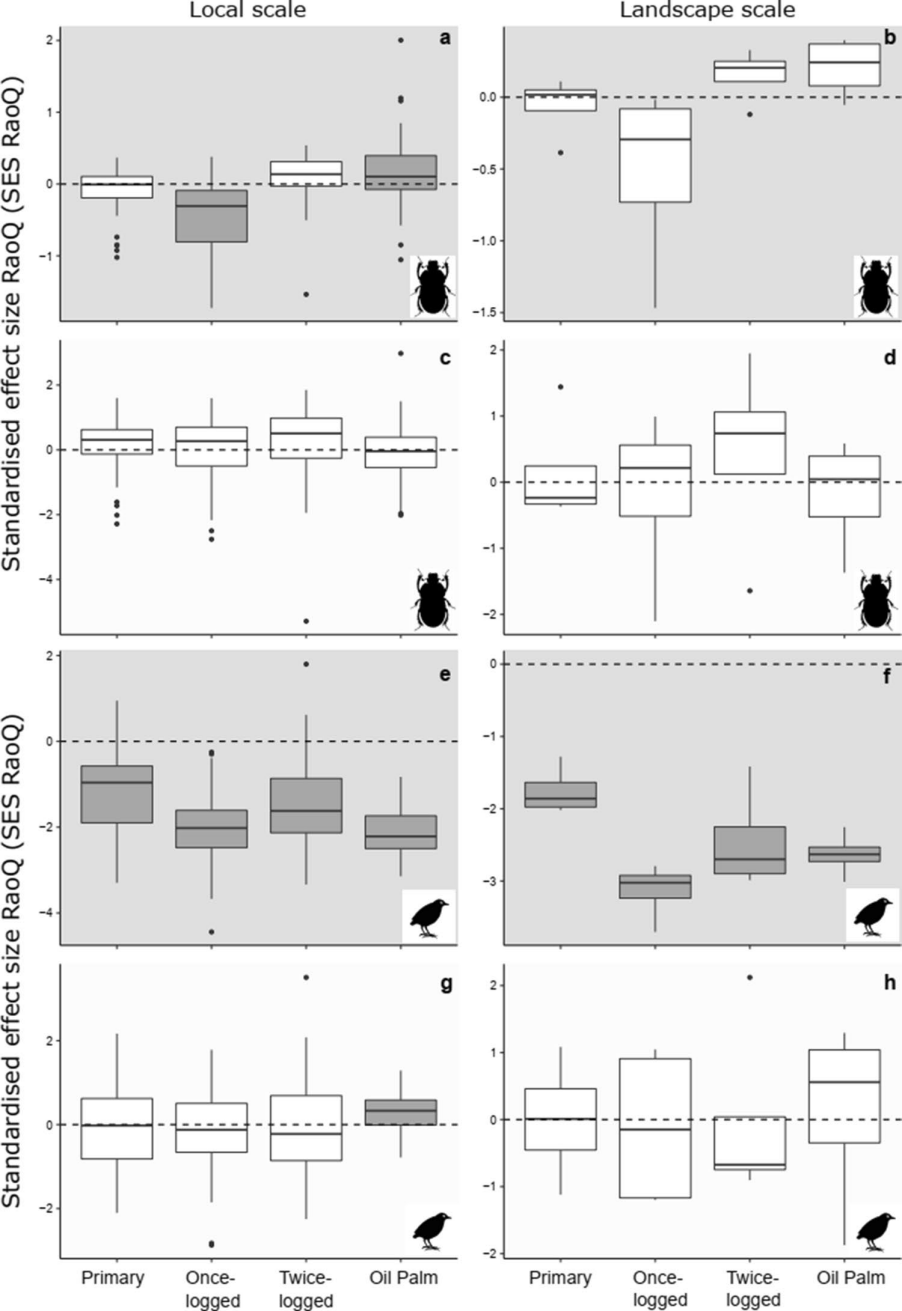
Variation in trait-based assembly for entire communities detected using the standardised effect size of RaoQ index (SES_RaoQ_) across four habitat types at local and landscape scales in Malaysian Borneo for dung beetles (**a**–**d**) and birds (**e**–**h**). Two null models were used to assess assembly patterns, grey-shaded backgrounds of plots refer to *model 1* (**a**, **b**, **e**–**f**), and white shaded backgrounds of plots refer to *model 2* (**c**, d, **g**–**h**). Grey boxplots denote SES values significantly different from zero, indicating non-random trait assembly, where trait convergence < 0 or trait divergence > 0. SES values that are not significantly different from zero are denoted by white boxplots, indicating random trait assembly

We sampled 65 dung beetle species comprising 26,285 individuals and 208 bird species across 7099 individual observations. When evaluating the assembly pattern of functional traits relative to communities where abundances of co-occurring species were randomised within a sample (*model 1*), at the local scale dung beetle communities varied considerably. Random trait assembly was found in primary and twice-logged forests, while trends in once-logged forest indicated trait convergence (*t*-test: *P* < 0.01) and in oil palm trait divergence (*t*-test: *P* < 0.01) (Fig. 1a, Online Resource 7). At the landscape scale, all dung beetle communities showed random trait assembly (Fig. 1b, Online Resource 7). The mean SES_RaoQ_ was consistently significantly lower than zero indicating trait convergence for bird communities in all habitats at both local scales (*t*-test: *P* < 0.01, Fig. 1e, Online Resource 7) and landscape scales (*t*-test: *P* < 0.01, Fig. 1f, Online Resource 7).

When evaluating the assembly pattern of functional traits relative to randomised abundances of species across samples (*model 2*), we found no evidence of non-random trait assembly for dung beetle communities at either spatial scales (*t*-test: *P* > 0.33, Fig. 1c, d, Online Resource 7). Following forest conversion to oil palm, trait divergence was identified (*t*-test: *P* < 0.01, Fig. 1g, Online Resource 7) in bird communities at the local scale, suggesting competition is influential. At the landscape scale, we found no evidence of non-random trait assembly for bird communities (*t*-test: *P* > 0.86, Fig. 1h, Online Resource 7).

### Co-occurrence in relation to habitat association

**Fig. 2:**
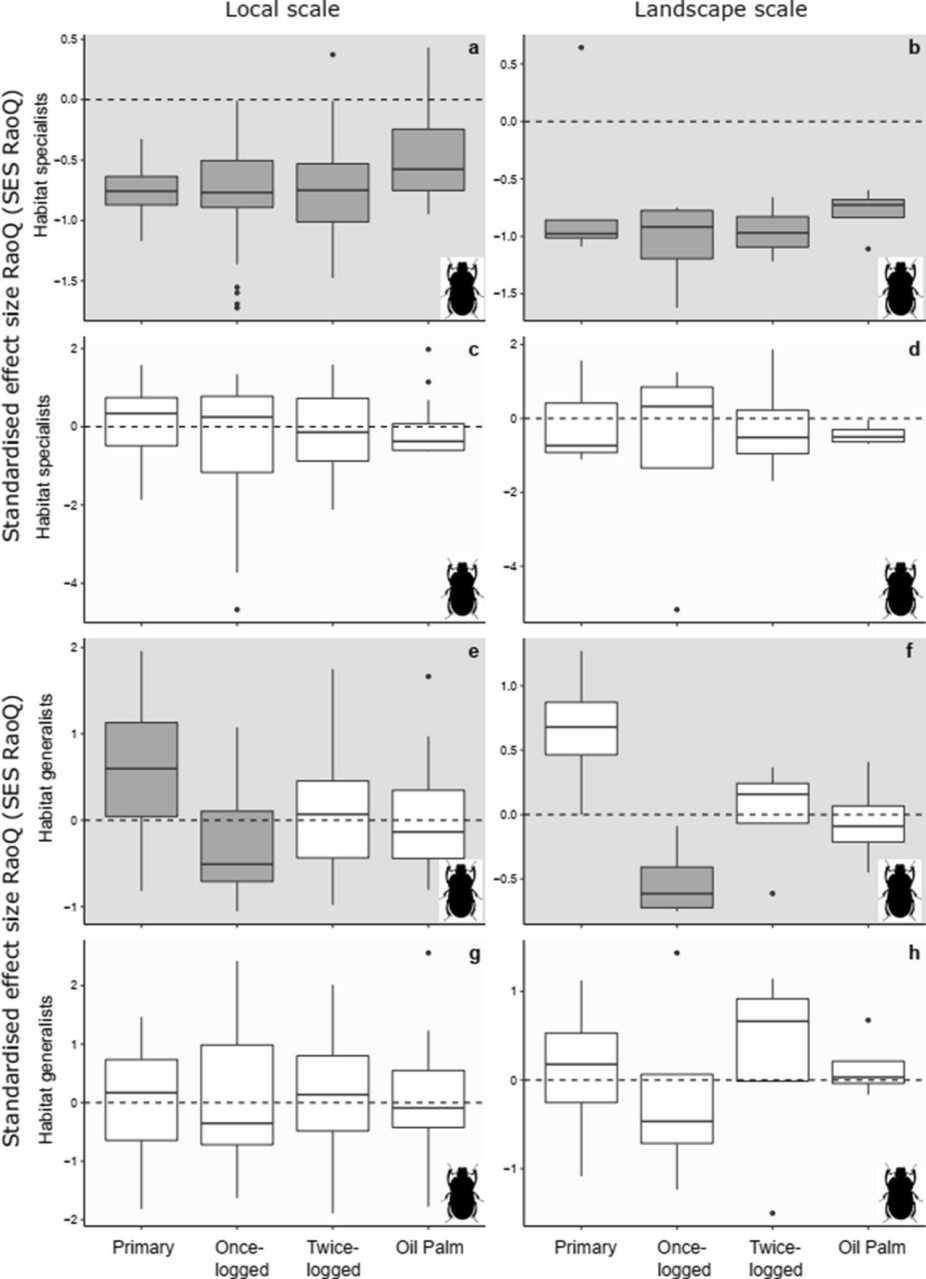
Variation in trait-based assembly for communities comprised of habitat specialists [species unique to oil palm or forests, (**a**–**d**)] and habitat generalist species (species found across all habitat types, **e**–**h**), detected using the standardised effect size of RaoQ index (SES_RaoQ_), across four habitat types at local and landscape scales in Malaysian Borneo for dung beetle communities. Two null models were used to assess assembly patterns, grey-shaded backgrounds of plots refer to *model 1* (**a**, **b**, **e, f**), and white shaded backgrounds of plots refer to *model 2* (**c**, **d**, **g, h**). Grey boxplots denote SES values significantly different from zero, indicating non-random trait assembly trait convergence < 0 or trait divergence > 0. SES values that are not significantly different from zero are denoted by white boxplots, indicating random trait assembly

For dung beetle habitat specialists, trait convergence was observed using *model 1* at both spatial scales (*t*-test: *P* < 0.01, Fig. 2a, b, Online Resource 8), suggesting environmental filtering is a key assembly mechanism. However, dung beetle habitat generalists using *model 1* revealed non-random assembly patterns in less degraded habitats, with trait divergence in primary forest at the local scale (indicating importance of competition; Fig. 2e) and trait convergence in once-logged forest at both local and landscape scales (indicating importance of environmental filtering, Fig. 2e, f). Contrastingly, in the most disturbed habitats (i.e., twice-logged forest and oil palm), trait-based assembly did not differ from random at both local and landscape scales (Fig. 2e–h). These patterns highlight shifts in assembly mechanisms between species of different habitat associations within the same habitat: in particular, habitat specialists in primary forest indicate trait convergence, while habitat generalists indicate trait divergence (Fig. 2). Analyses using *model 2* indicated trait assembly patterns that did not differ from random for both habitat specialists and generalists across all habitats and both spatial scales (*t*-test: *P* > 0.06 for all comparisons, Fig. 2c, d, g, h, Online Resource 8).

**Fig. 3:**
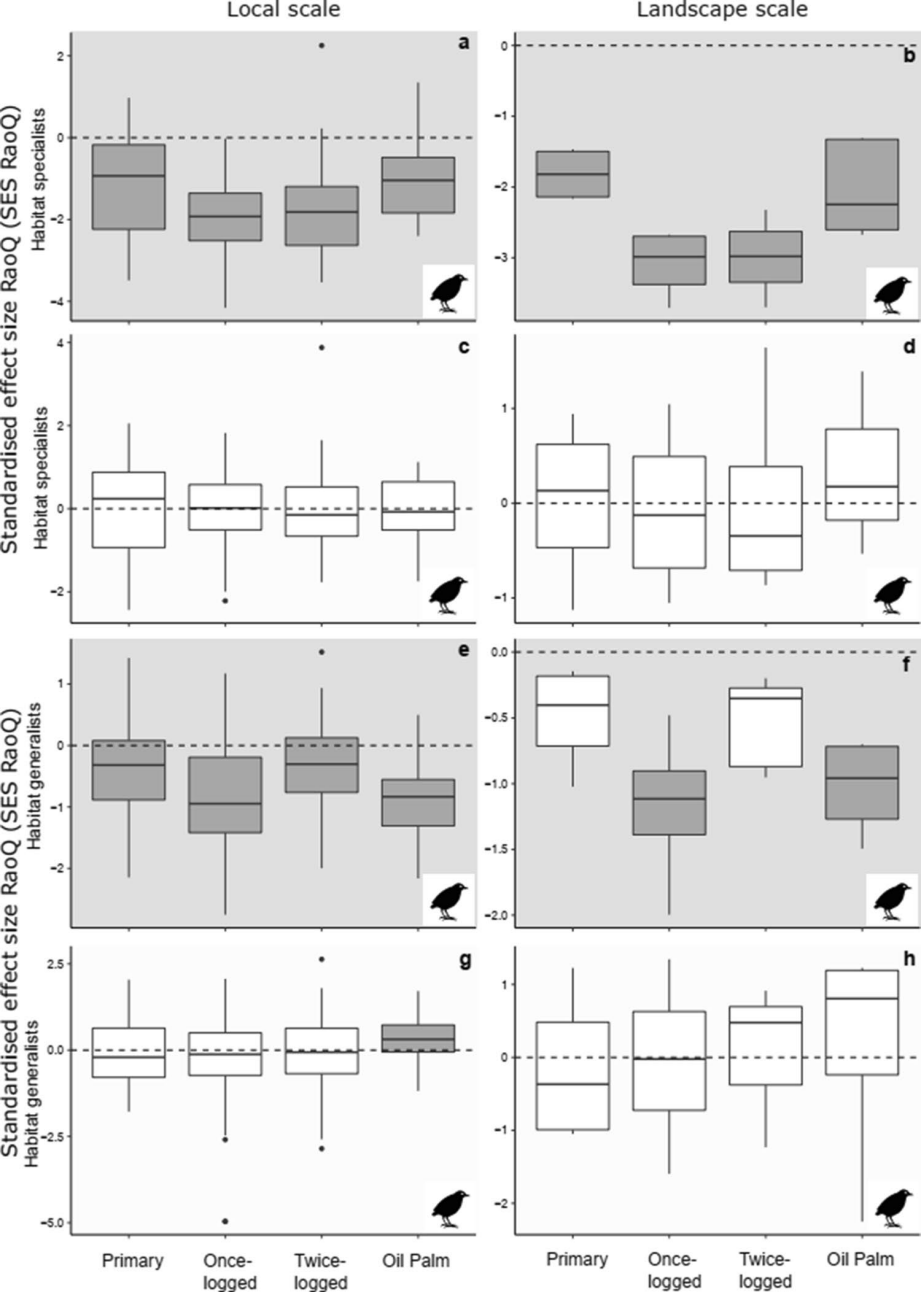
Variation in trait-based assembly, for communities comprised of habitat specialists (species unique to oil palm or forests, **a**–**d**) and habitat generalist species (species found across all habitat types, **e**–**h**), detected using the standardised effect size of RaoQ index (SES_RaoQ_), across four habitat types at local and landscape scales in Malaysian Borneo for bird communities. Two null models were used to assess assembly patterns, grey-shaded backgrounds of plots refer to *model 1* (**a**, **b**, **e**, **f**), and white shaded backgrounds of plots refer to *model 2* (**c**, **d**, **g**, **h**). Grey boxplots denote SES values significantly different from zero, indicating non-random trait assembly trait convergence < 0 or trait divergence > 0. SES values that are not significantly different from zero are denoted by white boxplots, indicating random trait assembly

Bird habitat specialists across habitats and spatial scales indicated trait convergence using *model 1*, while assembly patterns did not differ from random under *model 2* (Fig. 3a–d, Online resource 8). Bird habitat generalists also identified trait convergence in all habitats at the local scale, as well as in once-logged forest and oil palm at the landscape scale using *model 1* (*t*-test: *P* < 0.01, Fig. 3e, f, Online resource 8). However, using *model 2*, trait convergence was only observed in oil palm at the local scale (*t*-test: *P* < 0.01, Fig. 3g, Online resource 8) which is comparable to the overall community results (Fig. 1g, h).

### Co-occurrence in relation to nesting and foraging guilds

**Fig. 4:**
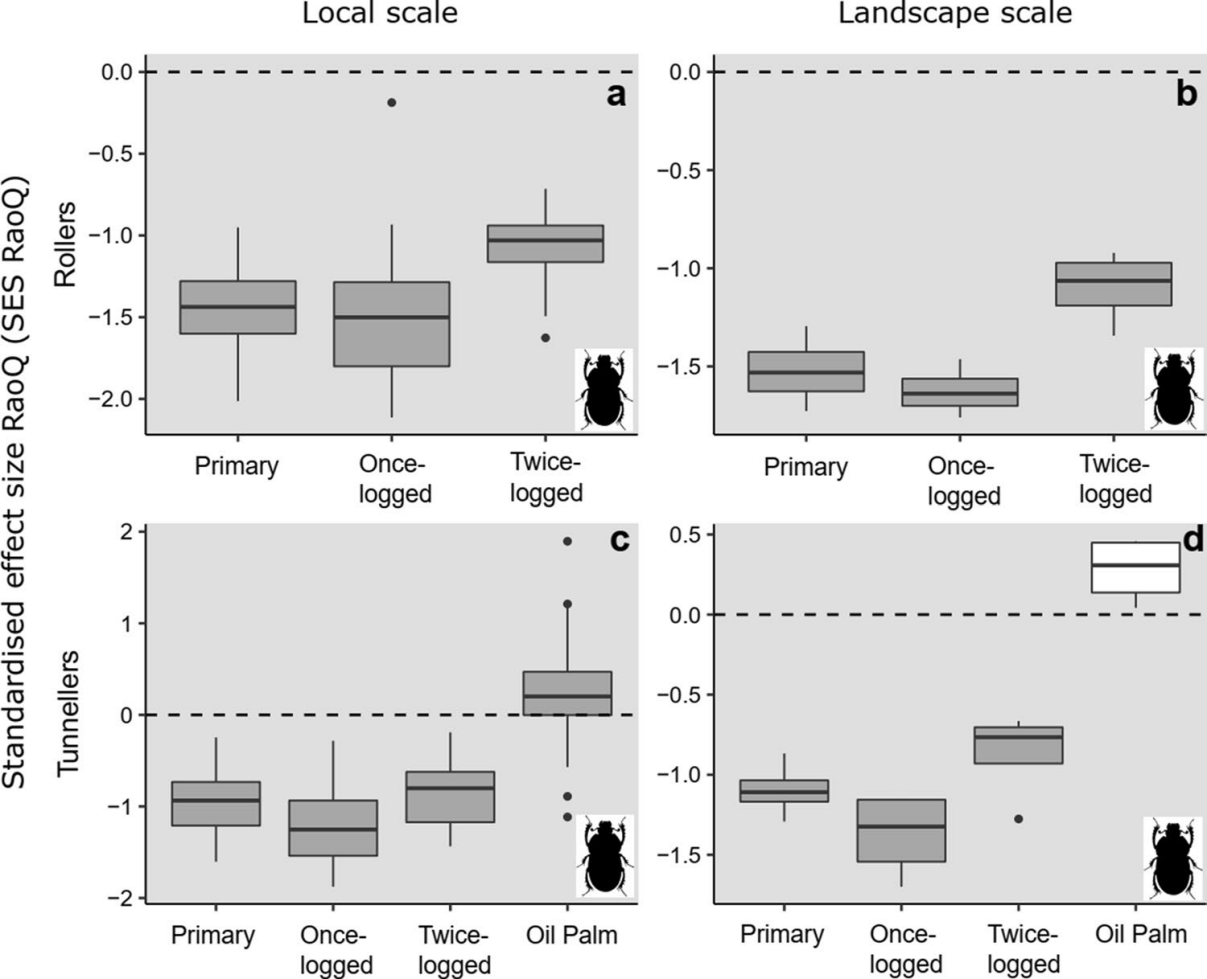
Variation in trait-based assembly, detected using the standardised effect size of RaoQ index (SES_RaoQ_), for dung beetle communities comprised of dominant nesting guilds (rollers—**a**, **b**, tunnellers—**c, d**) found across all habitat types, at local and landscape scales, in Malaysian Borneo. Rollers are absent in oil palm and are, therefore, not represented. Grey boxplots denote SES values significantly different from zero, indicating non-random trait assembly trait convergence < 0 or trait divergence > 0. SES values that are not significantly different from zero are denoted by white boxplots, indicating random trait assembly. Results are presented from *model 1* analyses

Nesting guilds of dung beetles indicated uniform trait-based assembly across habitats for rollers, which displayed trait convergence at both scales using *model 1* (Fig. 4a, b, Online Resource 10). Tunnelling species within oil palm communities displayed trait divergence at the local scale using *model 1* (*t*-test: *P* < 0.01, Fig. 4c), with a trend towards trait divergence at the landscape scale (*t*-test: *P* = 0.07, Fig. 4d, Online Resource 10). Forest tunnellers indicated trait convergence at both scales (Fig. 4c, d), implying a strong influence of environmental filtering. Assembly patterns did not differ from random across habitats or scales using *model 2* (Online Resource 10).

**Fig. 5:**
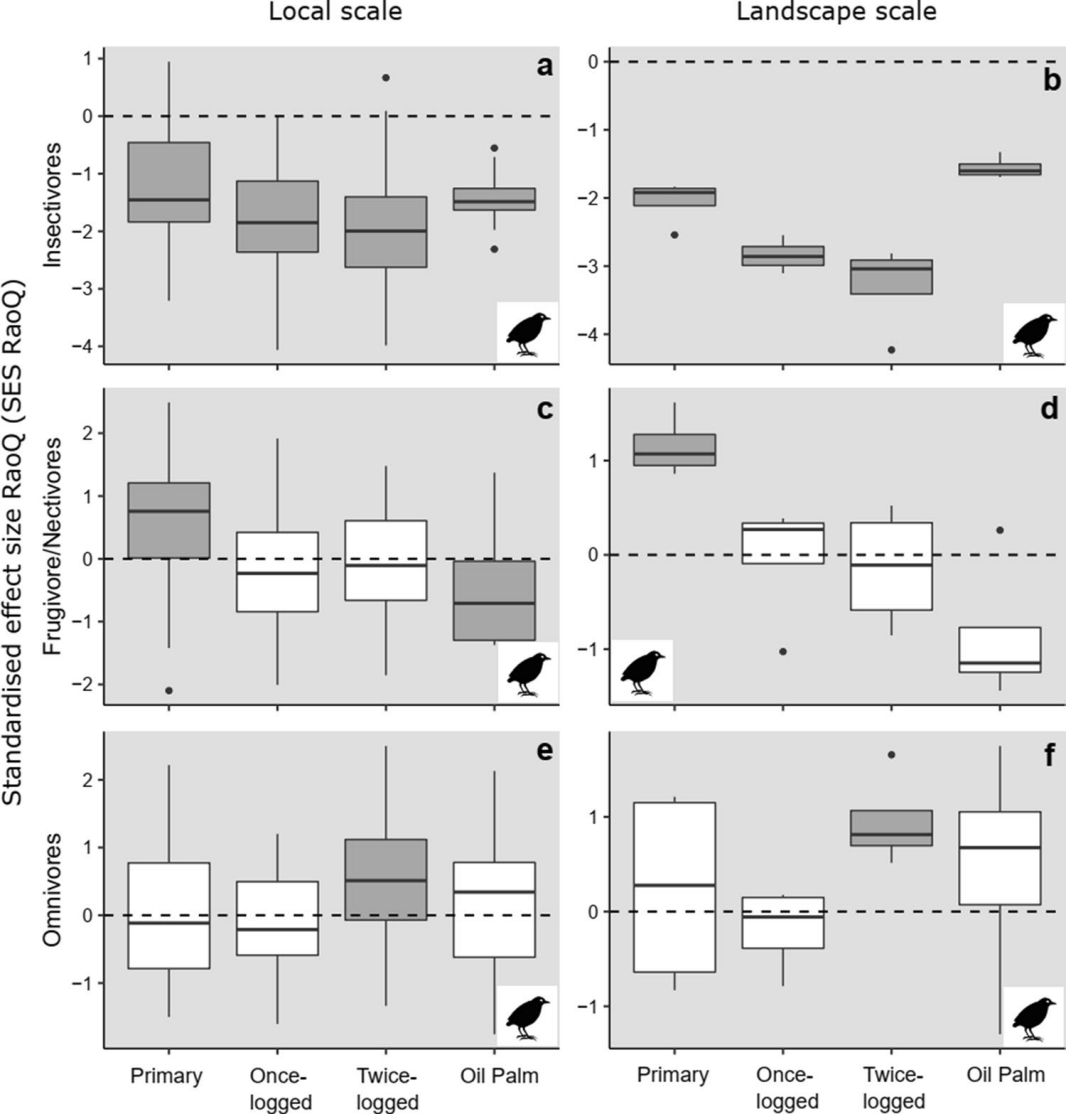
Variation in trait-based assembly for communities comprised of dominant feeding guilds (insectivores—**a**, **b**, frugivore/nectivores—**c**, **d**, omnivores—**e**, **f**) for bird species found across all habitat types, detected using the standardised effect size of RaoQ index (SES_RaoQ_), at local and landscape scales in Malaysian Borneo. Grey boxplots denote SES values significantly different from zero, indicating non-random trait assembly trait convergence < 0 or trait divergence > 0. SES values that are not significantly different from zero are denoted by white boxplots, indicating random trait assembly. Results are presented from *model 1* analyses

Dominant avian feeding guilds showed variation in trait-based assembly patterns using *model 1*. Insectivore communities indicated strong trait convergence across all habitats and scales (*t*-test: *P* < 0.01, Fig. 5a, b, Online Resource 10), mirroring overall community patterns (Fig. 1e, f). In contrast, frugivore/nectivores showed a gradient of change: primary forests displayed trait divergence at both scales (*t*-test: *P* < 0.01, Fig. 5c, d), logged forests did not differ from random (*t*-test: *P* > 0.20, Fig. 5c, d), and oil palm revealed trait convergence at the local scale (*t*-test: *P* < 0.01, Fig. 5c, d, Online Resource 10). In more disturbed habitats, omnivorous species indicated trait divergence at both scales, particularly in twice-logged forest (*t*-test: *P* < 0.01; Fig. 5e, f). Assembly patterns did not differ from random across habitats or scales using *model 2,* with the exception of insectivores in oil palm, which indicated trait divergence (Online Resource 10).

## Discussion

We explored the manner in which tropical land-use change influences species co-occurrence patterns to infer community assembly mechanisms in two indicator taxa. To our knowledge, this is the first assessment of dung beetle co-occurrence patterns, and the first multi-taxon, multi-spatial scale analysis of co-occurrence in relation to anthropogenic habitat change. In revealing evidence of non-random assembly at local scales in dung beetles, and variation between trait convergence and random assembly in birds across the disturbance gradient (Fig. 1e–h), our study highlights the sensitivity of community assembly mechanisms to anthropogenic disturbances, via a shift in the relative contribution of stochastic and deterministic processes. Our study also indicates important differences in how functional groups of species co-occur, indicating that whole-community analyses can mask critical findings.

### Community assembly and land-use

Our results varied considerably with the null model used for both taxa, providing field-based evidence for expectations of theoretical community assembly (de Bello et al. 2013). *Model 1,* which can detect limiting similarity, varied across the disturbance gradient, whereas *model 2,* which reliably detects environmental filtering (Götzenburger et al. 2016), suggested community assembly did not differ from random. Assembly patterns at the two spatial scales (i.e. 100 m and 10 s km) are very similar—with differences congruent with lower statistical power at larger scales—indicating that the mechanisms detected are robust to impacts of community turnover (Socolar et al. 2016). However, while we show similar impacts of land-use change on assembly patterns between taxa, as also observed for species richness and composition impacts (Barlow et al. 2007; Edwards et al. 2014a), we also reveal important variations. This underscores the importance of multi-taxon assessments for ecological understanding and conservation effectiveness.

We observed no impact of selective logging on bird community assembly, with trait convergence across primary and logged forests at both scales under *model 1* (Fig. 1e), suggesting environmental filtering (Grime 2006; Kraft and Ackerly 2010), since only one of our five traits (body size) could conceivably drive competitive dominance (sensu Mayfield and Levine 2010). However, logging once (but not twice) switched trait assembly from random to trait convergence in dung beetle communities, at the local scale under *model 1.* Minimally logged forests in Borneo reveal trait clustering in understory plants, although increased logging intensity decreased the influence of environmental filters (Döbert et al. 2017). Likewise, dung beetle communities in restored forests in Brazil showed a strong importance of environmental conditions (relative to space and landscape context) suggesting environmental filtering in these recovering forests (Audino et al. 2017).

The conversion of forest to oil palm showed evidence of changing assembly patterns in dung beetle (*model 1*; Fig. 1a) and bird (*model 2*; Fig. 1g) communities indicating trait divergence, and thus potentially an influence of competition, at the local scale. This supports previous studies revealing that non-random assembly processes prevail in harsher, stressed, or disturbed environments (Chase 2007), including bird communities in fragmented Atlantic Brazilian forests (Bregman et al. 2015) and tree communities in frequently burned African savanna (van der Plas et al. 2015). However, it contradicts theory suggesting that disturbance drives stronger environmental filtering as resources are homogenised (Kraft and Ackerly 2010; Mori et al. 2018; Wearn et al. 2018). Lower mammal population densities in oil palm (Wearn et al. 2017) suggest that dung resources are sparse, potentially increasing competitive influences on species co-occurrence. Likewise, the uniformity of oil palm likely reduces bird nesting opportunities, protection, and resource availability, potentially driving competition.

### Community assembly and functional variation

Assembly patterns for the overall community could potentially mask variation between sub-sets of a community determined by different functional traits or environmental needs (Bergman et al. 2015). Our results reveal how this is possible both within and across habitats. Within primary forests, opposing assembly patterns of forest specialists (trait convergence; Fig. 2a) and habitat generalists (trait divergence; Fig. 2e) underpin random assembly in the overall dung beetle community (Fig. 1a). Between habitats, mechanisms driving community assembly of habitat generalists differ with the intensity of disturbance for both taxa (Fig. 2e, f, Fig. 3g), while avian habitat specialists show strong trait convergence (i.e. large negative SES_RaoQ_ values, Fig. 3b) mirroring the overall community assembly patterns (Fig. 1f). In combination, this indicates that within pristine and disturbed habitats, patterns in overall communities are driven by both generalists and specialists, including rare species (Mori et al. 2018). Thus, solely taking a community-level approach is misleading, underscoring the need for more nuanced analysis of co-occurrence patterns (Bergman et al. 2015).

Despite declines in avian insectivores following logging and conversion to oil palm (Edwards et al. 2013; Hamer et al. 2015; Powell et al. 2015), this guild continues to dominate communities and interactions between insectivorous species play an influential role in community assembly after land-use change (Fig. 5). The importance of insectivores was also observed in some primary forest samples (note the spread in SES_RaoQ_ values, Fig. 5a), likely reflecting the heterogeneous environment of pristine rainforests. Trait convergence in frugivore/nectivores (but not omnivores) in oil palm suggests that simplified vegetation limits microhabitats for nesting and protection, and food resources. However, the interplay between microhabitat structure, microclimates, and prey abundance, and the associated effects on species interactions is still not understood (Powell et al. 2015). Trait divergence in frugivore/nectivores in primary forest versus random assembly in logged forests suggests greater competition in the former, but the abundance of floral and fruit resources in successional scrubs and vines in the latter (Ansell et al. 2011).

The impacts of land-use change on community assembly mechanisms are apparent in the shift of tunnelling dung beetle species from trait convergence in forested habitats, as previously inferred in restored tropical forest (Audino et al. 2017), to trait divergence in oil palm (Fig. 4c, d; but see Perrone et al. 2017). In the absence of rollers, which represent the other key nesting guild (Slade et al. 2007), co-occurrence of tunnelling species in oil palm is driven by competition rather than environmental filtering. Thus, our results across taxa again reveal hidden assembly mechanisms, and we suggest that exploring the link between dominance, beta-diversity and multifunctionality (Slade et al. 2017; Mori et al. 2018) is a critical future direction for improving understanding of how species interact across modified landscapes.

## Conclusion

This study identifies, for the first time, trait-based assembly mechanisms that structure the high biodiversity of tropical rainforests, and how they are impacted by habitat modification (selective logging) and conversion (to oil palm plantations). Critically, we highlight the potential hidden effects of land-use change beyond altered community structure (Cardinale et al. 2002; Royan et al. 2016) and identify species co-occurrence patterns that are obscured when only a full community approach is considered. Taken together, our results underscore the importance of logged forests as refugia for biodiversity (Edwards et al. 2014a), but also indicate that communities are under intense competition for resources within oil palm, with unknown consequences for species persistence following further environmental stresses (e.g. El Niño drought or intensified management). Further research examining contributing factors, such as phylogenetics, physiology, and micro-habitat relationships (Boulangeat et al. 2012; Fernandez-Fournier et al. 2018; Start et al. 2018), would further clarify the extent and relative contribution of assembly mechanisms in these complex tropical ecosystems. Additionally, exploring the overlap between community assembly and ecosystem functioning is of key importance for improved ecological understanding of land-use change.

## Supplementary Information

Below is the link to the electronic supplementary material.

Supplementary file1 (DOCX 1586 KB)
