# Supplementary material for: Tropical land-use change alters trait-based community assembly rules for dung beetles and birds
Source: Oecologia. 2021 Feb 8;195(3):705–17. doi: 10.1007/s00442-020-04829-z (PMC7940334; doi:10.1007/s00442-020-04829-z)

**SUPPLEMENTARY ONLINE MATERIAL - Oecologia**

**Tropical land-use change alters trait-based community assembly rules for dung beetles and birds**

**Felicity A. Edwards^1,2*^, David P. Edwards^2^, Keith C. Hamer^1^, Tom M. Fayle^3,4^**

^1^ *School of Biology, University of Leeds, Leeds, LS2 9JT, UK.*

^2^ *Department of Animal and Plant Sciences, University of Sheffield, Sheffield, S10 2TN, UK.*

^3^ *Biology Centre of the Czech Academy of Sciences, Institute of Entomology Ceske Budejovice, 370 05, Czech Republic.*

^4^ *Institute of Tropical Biology and Conservation, Universiti Malaysia Sabah, Kota Kinabalu, Sabah, Malaysia*

** Address correspondence to F. A. Edwards, email* [*felicity.edwards@sheffield.ac.uk*](mailto:felicity.edwards@sheffield.ac.uk)

**Table of Contents:**

**Online resource 1 -** Location of the study area and schematic showing the spatial scale of the sampling design.

**Online resource 2 –** Description of functional traits.

**Online resource 3 –** Observed RaoQ values for the overall community.

**Online resource 4 –** Observed RaoQ values for species grouped by habitat associations with the full community and singletons removed.

**Online resource 5 –** Observed RaoQ values for dung beetle species grouped by nesting guilds

**Online resource 6 –** Observed RaoQ values for bird species grouped by foraging guilds

**Online resource 7 –** Null model outputs for overall community assembly.

**Online resource 8 –** Null model outputs for community assembly of species determined by habitat associations.

**Online resource 9 –** Null model outputs for community assembly of species determined by habitat associations, with the removal of singletons.

**Online resource 10 –** Null model outputs for community assembly of species determined by nesting and foraging guilds.

**
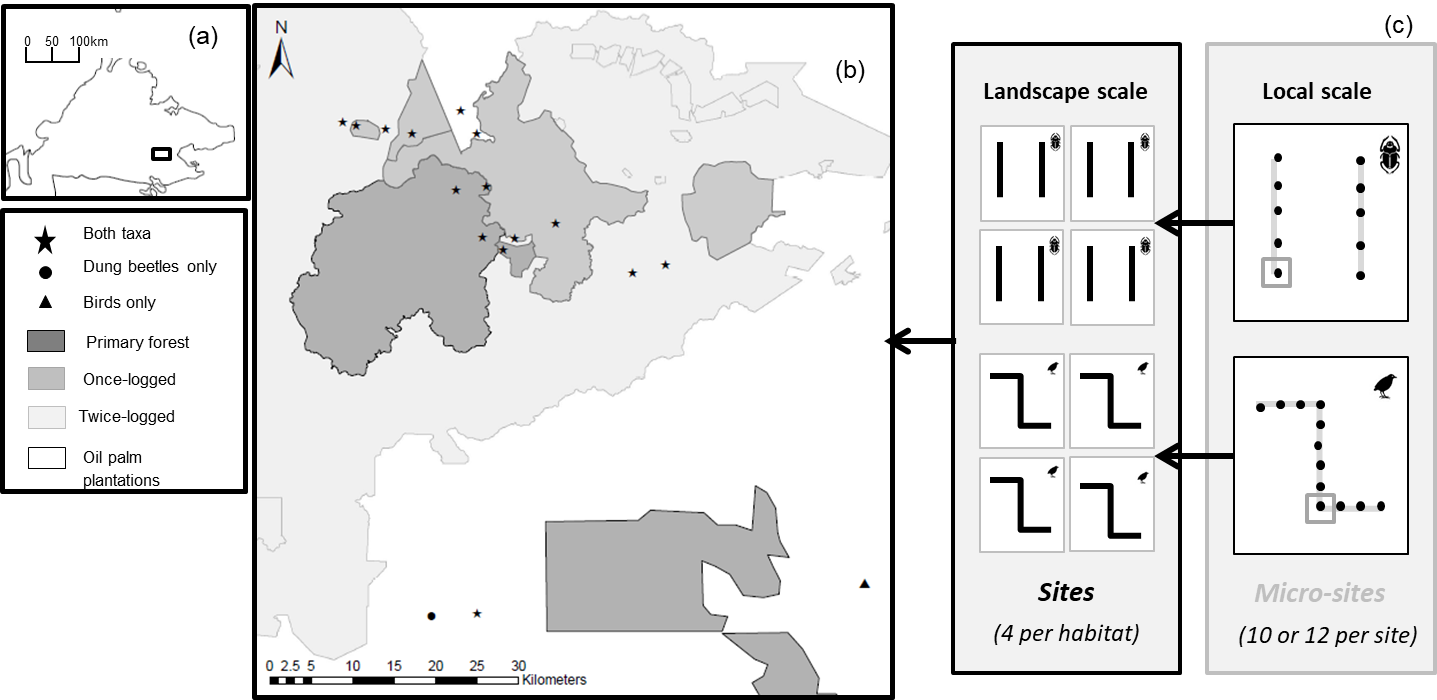
**

**Online resource 1:** a) Location of the study area box) in Sabah, Borneo. b) A map of the study area. The symbols on the map identify the sampling sites (n=17) at which dung beetles (black circle, n=1), birds (black triangle, n=1) or both taxa (black star, n=15) were sampled. In all cases, the mid-point of the two transects at a given site is represented on the map. c) Schematic showing the spatial scale of the sampling design. Within the overall study landscape sampling occurred across four habitats at the 1) LOCAL scale, with 10 dung beetle traps or 12 bird point counts, comprising the microsites at each site, and 2) LANDSCAPE scale, composed of four sites for each habitat type, denoted by symbols in the inset map (b).

**Online resource 2:** Description of dung beetle and bird functional traits used in the functional diversity analyses which form the basis of the null model randomisation analyses.

| Taxa | Trait | Measure | Description |
| --- | --- | --- | --- |
| Dung beetles | Body size | Continuous | Elytra width x Pronotum and elytra length |
|  | Guild | Factor | Roller, Tunneller or Dweller |
|  | Diel activity | Binary | Diurnal or Nocturnal |
|  | Diet substrates | Multi-choice binary | Dung, Carrion, Fruit & Fungi |
|  | Common diet substrate | Factor | Dung, Carrion, Fruit or Fungi |
| Birds | Body size | Continuous | Body length |
|  | Guild | Multi-choice binary | Insectivore, Frugivore, Grainivore, Piscivore, Predator, Nectivore, Scavenger |
|  | Foraging mode | Multi-choice binary | Sallying, Gleaning, Probe/Dig, Pursuit |
|  | Foraging substrate | Multi-choice binary | Air, Water, Vegetation, Arboreal, Ground |
|  | Bill shape | Factor | Straight, decurved, hooked |

**Online resource 3:** Variation in observed RaoQ for the entire community across four habitat types for dung beetles (a) and birds (b), at landscape (site level analysis, coral) and local (trap level analysis, blue) scales in Malaysian Borneo.


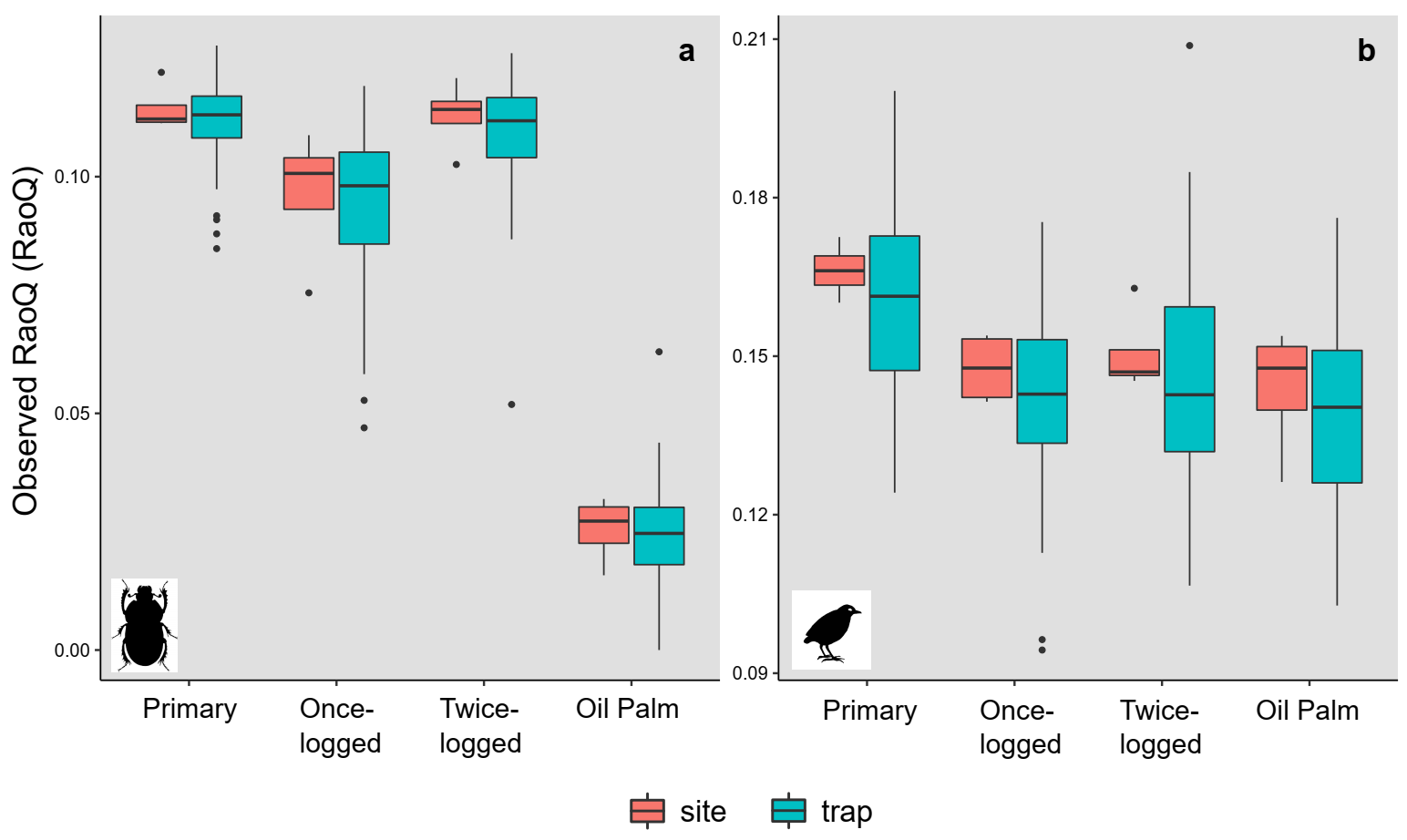


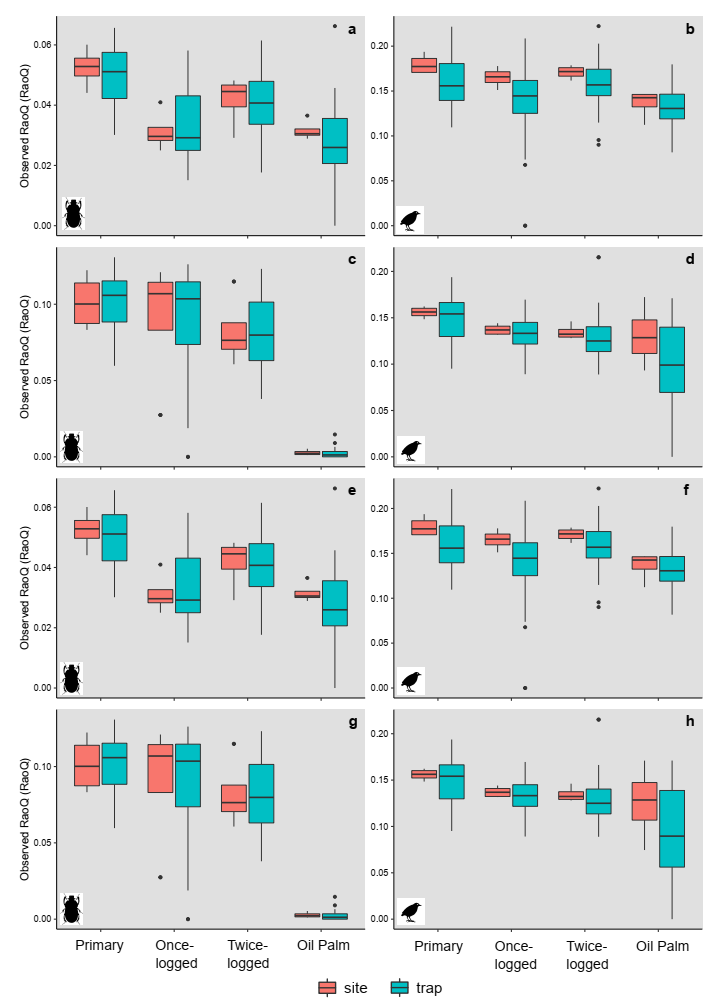
**Online resource 4:** Variation in observed RaoQ for the entire community (a-d) and with singletons removed (e-h), for species grouped by habitat associations. Habitat associations were defined as habitat specialists (species unique to oil palm or forests, [a-b, e-f]) or habitat generalists (species found across all habitat types, c-d, g-h). These are considered across four habitat types at both landscape (site level analysis, coral) and local (trap level analysis, blue) scales in Malaysian Borneo for dung beetle and bird communities.

**Online resource 5:** Variation in observed RaoQ for the entire dung beetle community, for species grouped by the two principal nesting guilds, rollers (a) and tunnellers (b), across four habitat types at both landscape (site level analysis, coral) and local (trap level analysis, blue) scales in Malaysian Borneo. Note rollers were absent in oil palm and are therefore not presented in figure a.


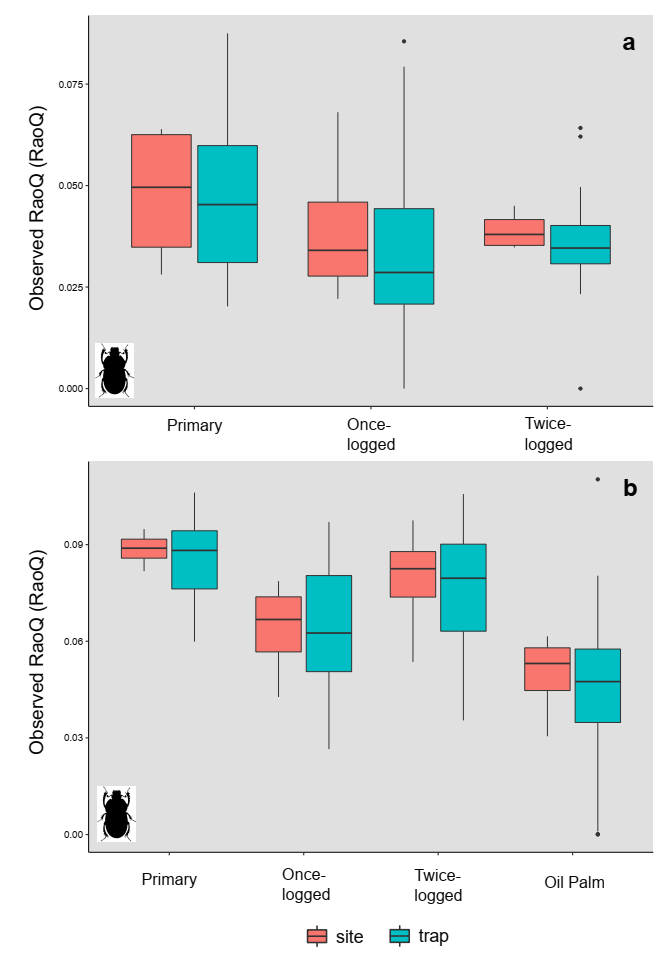


**Online resource 6:** Variation in observed RaoQ for the entire bird community, for species grouped by three key foraging guilds, invertebrates (a), frugivores/nectivores (b), and omnivores (c), across four habitat types at both landscape (site level analysis, coral) and local (trap level analysis, blue) scales in Malaysian Borneo.


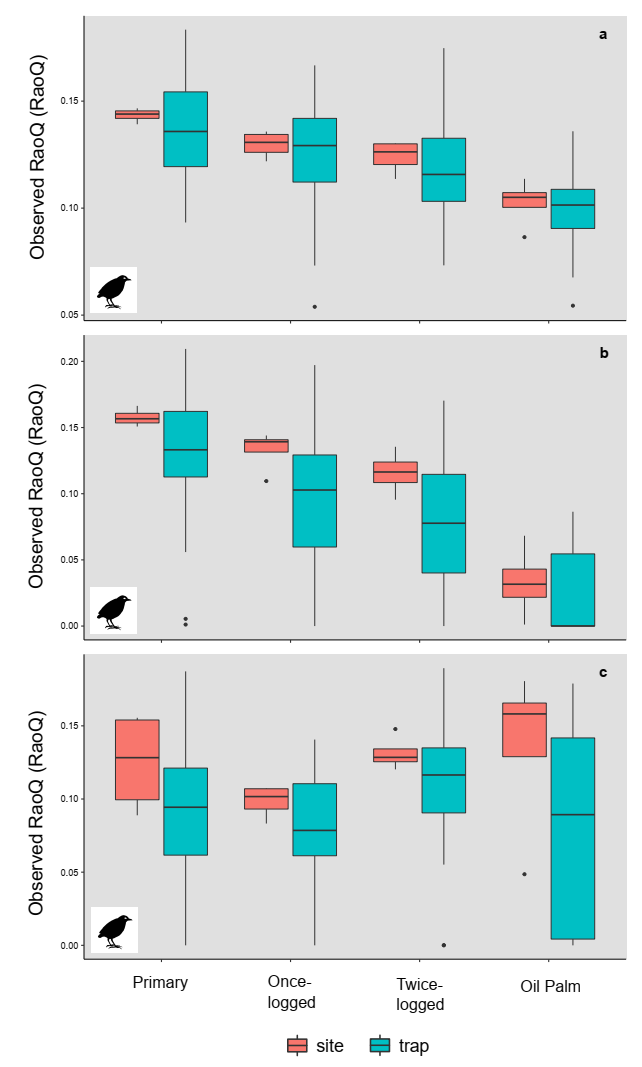


**Online resource 7:** Variation in community assembly using the standard effect size of the RaoQ index (SES_RaoQ_) analysed across four habitat types and two sampling scales (local = trap level analysis, and landscape = site level analysis), for dung beetles and birds in Malaysian Borneo. Analyses were conducted using the full community data. *P*-values were obtained from testing if the mean SES_RaoQ_ was significantly different from zero. Bold text represents significant results, grey shading defines null *model 1*, and white shading refers to the use of null *model 2*. These null models use different randomisation algorithms. Mean observed RaoQ values are also presented.


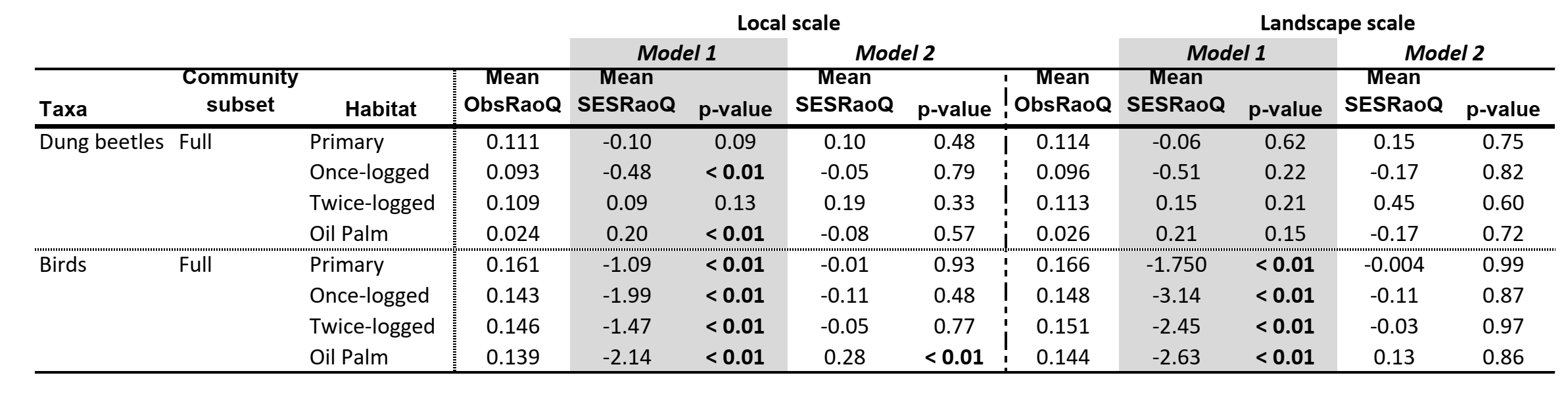


**Online resource 8:** Variation in community assembly using the standard effect size of the RaoQ index (SES_RaoQ_) analysed across four habitat types and two sampling scales (local = trap level analysis, and landscape = site level analysis), for dung beetles and birds in Malaysian Borneo. Analyses were conducted using the full community data across communities determined by habitat associations (*habitat generalists* - shared species between all forest types and oil palm, and *habitat specialists* –species unique to forest or oil palm. *P*-values were obtained from testing if the mean SES_RaoQ_ was significantly different from zero. Bold text represents significant results, grey shading defines null *model 1*, and white shading refers to the use of null *model 2*. These null models use different randomisation algorithms. Mean observed RaoQ values are also presented.


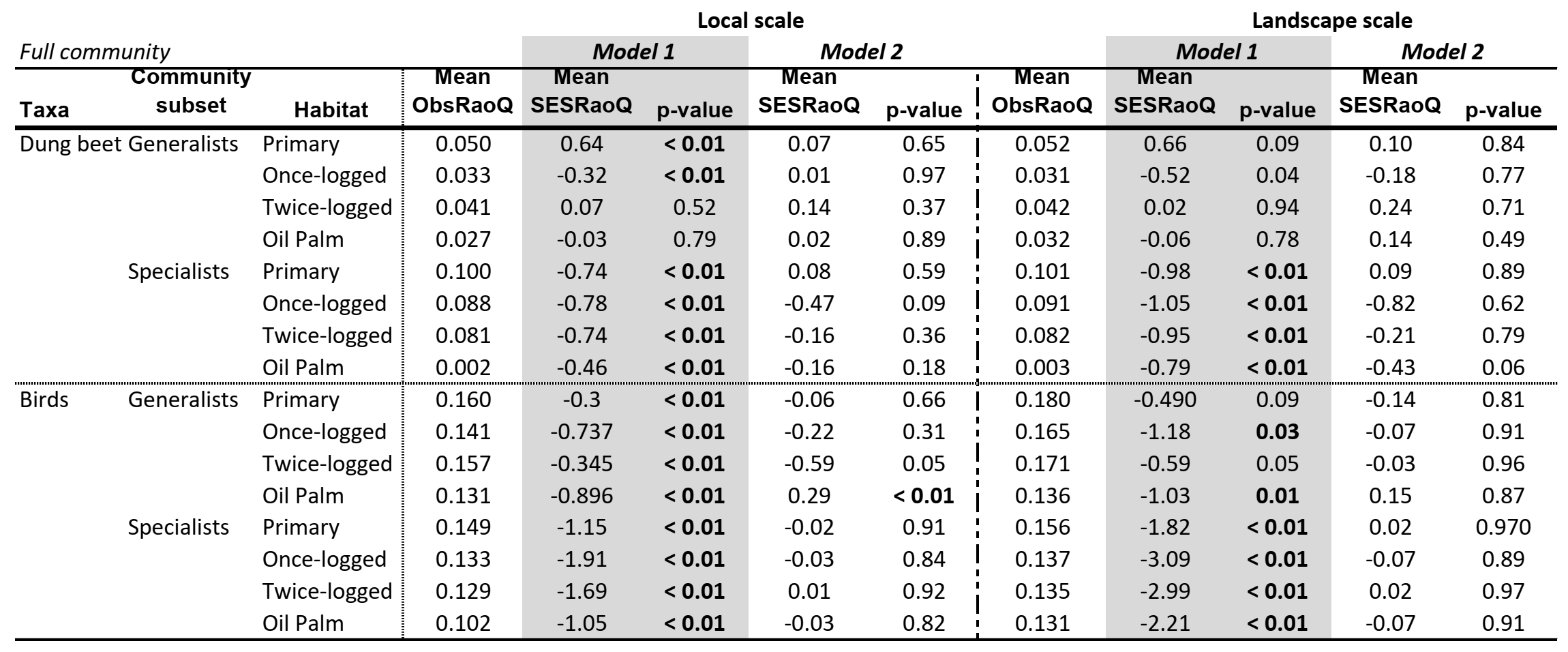


**Online resource 9:** Variation in community assembly using the standard effect size of the RaoQ index (SES_RaoQ_) analysed across four habitat types and two sampling scales (local = trap level analysis, and landscape = site level analysis), for dung beetles and birds in Malaysian Borneo. Analyses were conducted across communities with singletons removed. Communities were determined by habitat associations (*habitat generalists* - shared species between all forest types and oil palm, and *habitat specialists* –species unique to forest or oil palm. *P*-values were obtained from testing if the mean SES_RaoQ_ was significantly different from zero. Bold text represents significant results, grey shading defines null *model 1* used, and white shading refers to the use of null *model 2*. These null models use different algorithms. Mean observed RaoQ values are also presented.


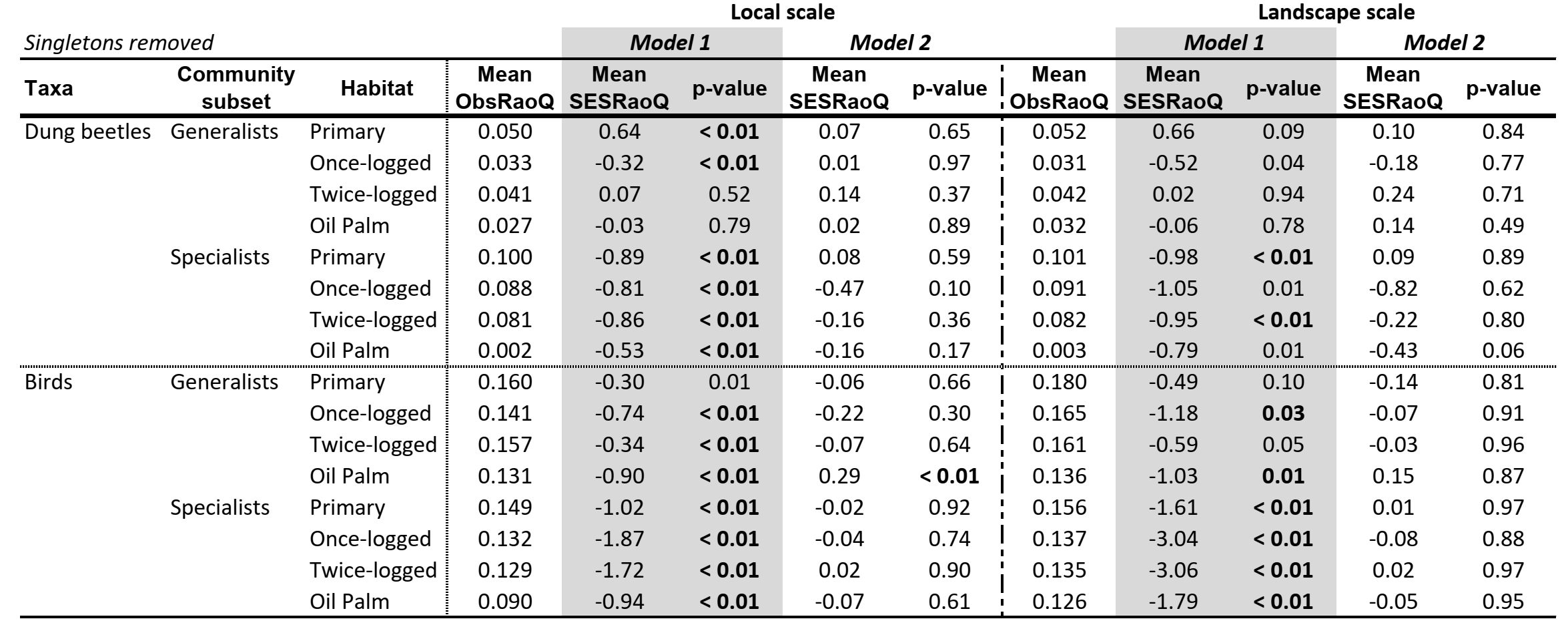


**Online resource 10:** Variation in community assembly using the standard effect size of the RaoQ index (SES_RaoQ_) analysed across four habitat types and two sampling scales (local = trap level analysis, and landscape = site level analysis), for dung beetles and birds in Malaysian Borneo. Analyses were conducted across communities determined by key nesting and foraging guilds. *P*-values were obtained from testing if the mean SES_RaoQ_ was significantly different from zero. *P*-values were obtained from testing if the mean SES_RaoQ_ was significantly different from zero. Bold text represents significant results, grey shading defines null *model 1*, and white shading refers to the use of null *model 2*. These null models use different algorithms. Mean observed RaoQ values are also presented.


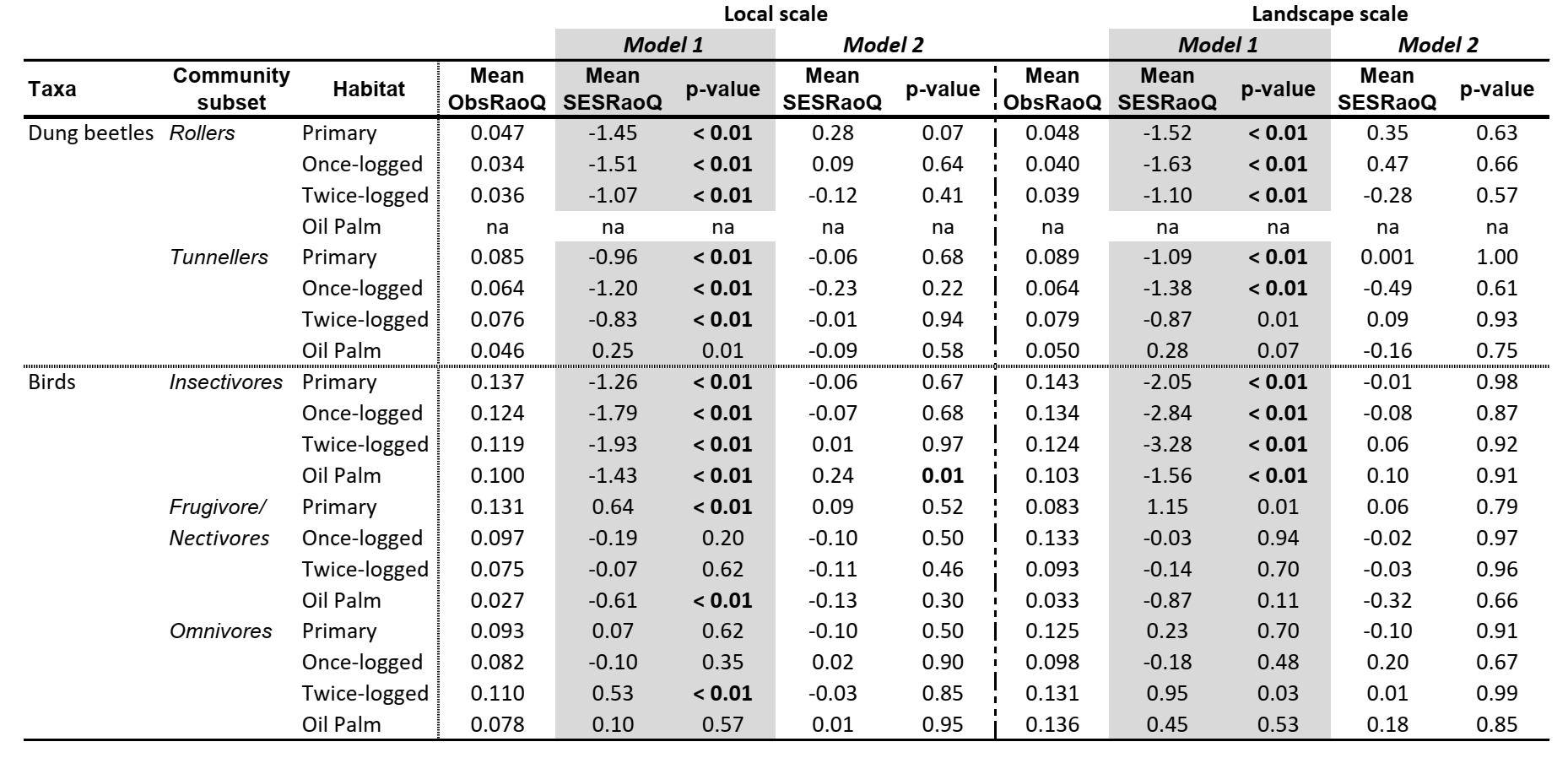

Supplement: Supplementary file 1 — Supplementary file1 (DOCX 1586 KB) [file 442_2020_4829_MOESM1_ESM.docx]
